# The power of phase II end-points for different possible mechanisms of action of an experimental treatment

**DOI:** 10.1016/j.ejca.2015.03.002

**Published:** 2015-05

**Authors:** J.M.S. Wason, A. Dentamaro, T.G. Eisen

**Affiliations:** a MRC Biostatistics Unit, Cambridge, United Kingdom; b Department of Brain and Behavioral Sciences, University of Pavia, Italy; c Cambridge Clinical Trials Centre, Cambridge Biomedical Research Centre, United Kingdom

**Keywords:** Measurement error, Phase II cancer trial, Progression-free-survival, RECIST, Response

## Abstract

**Background:**

The high failure rate in phase III oncology trials is partly because the signal obtained from phase II trials is often weak. Several papers have considered the appropriateness of various phase II end-points for individual trials, but there has not been a systematic comparison using simulated data to determine which end-point should be used in which situation.

**Methods:**

In this paper we carry out simulation studies to compare the power of several Response Evaluation Criteria in Solid Tumours (RECIST) response-based end-points for one-arm and two-arm trials, together with progression-free survival (PFS) and testing the tumour-shrinkage directly for two-arm trials. We consider six scenarios: (1) short-term cytotoxic therapy; (2) continuous cytotoxic therapy; (3 + 4) cytostatic therapy; (5 + 6) delayed tumour-shrinkage effect (seen in some immunotherapies). We also consider measurement error in the assessment of tumour size.

**Results:**

Measurement error affects the type-I error rate and power of single-arm trials, and the power of two-arm trials. Generally no single end-point performed well in all scenarios. Best observed response rate, PFS and directly testing the tumour-shrinkages performed best for a number of scenarios. PFS performed very poorly when the effect of the treatment was short-lived. In scenario 6, where the delay in effect was long, no end-point performed well.

**Conclusions:**

A clinician setting up a phase II trial should consider the likely mechanism of action the drug will have and choose an end-point that provides high power for that scenario. Testing the difference in tumour-shrinkage is often powerful. Alternative end-points are required for therapies with a long delayed effect.

## 1 Introduction

The strength of signal obtained in phase II oncology trials is often low and this partly accounts for the worryingly high failure rate of phase III oncology trials [1,2]. The gold-standard end-point in most tumour types is overall survival (OS). However OS events generally accrue too slowly to be used as the primary end-point in a phase II trial. OS is also affected by treatment crossover upon disease progression [3] In solid tumours, phase II end-points are generally based on the Response Evaluation Criteria in Solid Tumours (RECIST) [4]. RECIST categorises each patient by the effect of the treatment on their tumour lesions. At each time the tumour is measured, the patient is categorised as having a complete response (CR), partial response (PR), stable disease (SD) or progressive disease (PD).

There are two main types of end-points based on RECIST: binary and time-to-event. Binary end-points include the response rate (RR), the proportion of patients whose tumour exhibits a PR or CR, and the disease control rate (DCR), the proportion of patients whose tumour exhibits SD, PR or CR. Time-to-event end-points include progression-free-survival (PFS) and time-to-progression (TTP): the former measures time to progression or death and the latter treats death as a censoring event. RR is considered suitable for cytotoxic drugs but less suitable for cytostatic agents [5,6]. PFS is said to be more informative in this case [7]. Various critiques have been made of PFS [8–10], although many criticisms also apply to RR and DCR.

The binary end-points can be further divided into fixed (also known as landmark) or best observed. In fixed analyses the RECIST categorisation at a fixed time after treatment commences is used as the end-point. In assessing best observed response, each patient is followed up until the end of treatment, and most favourable observed RECIST categorisation is used as the end-point. Intuitively, best observed response might be thought to be more efficient as it makes use of patient measurements taken at all timepoints. However, in colorectal and lung cancer trials, fixed analyses were more informative for long-term survival than best observed response [11].

Some previous literature has compared various phase II end-points, e.g. [11–15]. However, these studies have been based on a small number of trials, and apply only to specific treatments and tumour types. The literature currently lacks a study that investigates which factors affect the power of different end-points. In this paper we carry out simulation studies comparing the RECIST-based end-points in terms of power of the phase II trial. We also consider the approach of testing the tumour shrinkage directly [16,17]. This will mean the most suitable phase II end-point can be chosen based on the hypothesised mechanism of treatment on OS. We consider both one-arm and randomised trials. For one-arm trials we only consider response-based end-points as the other two methods are not recommended [7,16]. An important contribution of this paper is an investigation of the effect that measurement error, which can be large [18], has on trials using the different end-points.

## 2 Methods

### 2.1 Notation

For a one-arm trial testing the response-rate (however response is defined) of the experimental treatment, the true unknown response rate is denoted as *p*. The null hypothesis tested is:

(1) $$H_{0}:p⩽p_{0}$$

At the end of the trial, the null hypothesis is rejected if the number of responders is above a critical value, chosen so that the type-I error rate of the trial (the probability of rejecting *H*_0_ when *p* = *p*_0_) is equal to some level, often 5% or 10%. The sample size is chosen so that the power to reject *H*_0_ exceeds some level such as 80% or 90% when the true value of p is equal to a clinically relevant effect, *p*_1_.

A randomised trial tests the difference in effectiveness between an experimental treatment and a control treatment. For response-based end-points, we denote the true response rates of the control and experimental treatments as p_0_ and p_1_ respectively. We formulate the null hypothesis in terms of the odds-ratio (OR), which is:

(2) $$\text{OR}=\frac{\left(\frac{p_{1}}{1-p_{1}}\right)}{\left(\frac{p_{0}}{1-p_{0}}\right).}$$

The null hypothesis is:

(3) $$H_{0}:\mathrm{OR}⩽1$$

In this case, *H*_0_ is rejected if the 95% confidence interval for OR does not include 1, giving a two-sided 5% significance level.

For PFS, we consider the null hypothesis being in terms of the hazard ratio (HR), the ratio of the hazard in the experimental arm to the hazard in the control arm. We assume proportional hazards, i.e. HR is constant over time. The null hypothesis to be tested would be:

(4) $$H_{0}:\mathrm{HR}⩾1\text{.}$$

In this case, *H*_0_ can be tested using the log-rank test, or using a Cox proportional-hazards model. We use a Cox model and reject *H*_0_ if the *p*-value for the treatment effect is below 0.05.

### 2.2 Simulation study

In order to compare the performance of the different end-points under different scenarios with known properties, we used simulation studies. Each scenario represents a different mechanism of action that a new treatment may have. In order to better understand which situations favour which end-point, we simulated separately the size of target tumour lesions for each patient at each visit and also whether they progressed for a non-growth reason (for example due to new lesions, or an unacceptable increase in non-target tumour lesions).

We provide a brief, less technical, overview of how the data were simulated. A full technical description is given in the supplementary material.

The target tumour lesion data were simulated assuming that the log tumour-size ratio [19] is normally distributed. The log tumour-size ratio at a follow-up time for a patient with total length of lesions *X*cm and previous observed length of lesions *Y*cm is:

(5) $$\log\left(\frac{X}{Y}\right)$$

For each patient, a baseline target lesion size is simulated from a uniform distribution between 1 and 10 cm. The log tumour-size ratio between two consecutive timepoints is simulated from a normal distribution. The mean of this normal distribution is allowed to be different for each follow-up time, depending on the scenario.

Measurement error is simulated to be normally distributed on the log-scale, with zero mean and a specified variance. As the variance increases, the potential range of measurement error increases. Further detail is provided in the supplementary material.

Once the true and observed tumour size at each time are simulated, the time (if any) at which the patient experienced a progression for non-growth reasons (i.e. any reason other than an increase in the target lesion size) was simulated. This was simulated using a logistic regression, where the probability of progressing due to non-growth reasons at each timepoint, is specified. Parameters that determined mean tumour shrinkage and probability of progressing for non-growth reasons were varied between the two treatments in the case of randomised trials. The type-I error rate was estimated by simulating data with no difference in the mean log tumour-size ratio or probability of non-growth failure between the experimental treatment and control treatment (for two-arm studies) or historical control (for one-arm studies).

For single-arm trials, a sample size of 30 was used; for two-arm trials, 150 (75 per arm) was used. These were chosen to represent typical sample sizes seen in practice. For each simulation, 50,000 trial replicates were used.

### 2.3 Response assessment

Given a patient’s simulated target lesion size and progression for non-growth reasons, the estimate of benefit as assessed by each of the different end-points can be calculated. For the two fixed response end-points (DCR and RR), we assume that the RECIST classification at the second follow-up timepoint is used to classify the treatment as having succeeded or failed, with the treatment also counted as failed if the patient’s tumour progressed at the first follow-up time point for other reasons. For the two best observed response end-points, the time of progression was calculated for each patient – this is the time that either the target lesion size was more than 20% increased compared to the nadir (i.e. the smallest size it had been so far) or a non-growth progression occurred. Each visit up to progression was classified according to the RECIST criteria. The most recent RECIST guidelines [4] state that for single-arm trials, the best observed response requires confirmation. Although confirmation is not stated to be required for two-arm trials, for simplicity we used the same definition in all cases. Thus, if two subsequent observations before progression were PR or CR (for RR) or SD, PR or CR (for DCR), the treatment was classified as a success, otherwise as a failure.

For PFS, the end-point was the time at which a patient progressed for any reason. If the patient’s tumour did not progress, in the follow-up time, then they were censored at their final observation time.

We also use the method of testing the difference in tumour shrinkage directly [16], which is done at the second time-point. This is described further in the supplementary material.

## 3 Results

### 3.1 Single-arm studies

Six main scenarios are described in Table 1, with more technical details in supplementary material. In all cases, baseline measurements and six follow-up times were considered. In the first scenario the experimental treatment provides a benefit in terms of tumour shrinkage for the first two follow-up times; the second is similar except the benefit is across all follow-up times; in the third, the experimental treatment provides a benefit by reducing the probability of non-growth progression; the fourth is a mixture of the second and third; in the fifth scenario, the experimental treatment provides a benefit in mean tumour shrinkage from the second follow-up time onward; the sixth scenario is similar to the fifth scenario but from the fourth follow-up time.

**Fig. 1:**
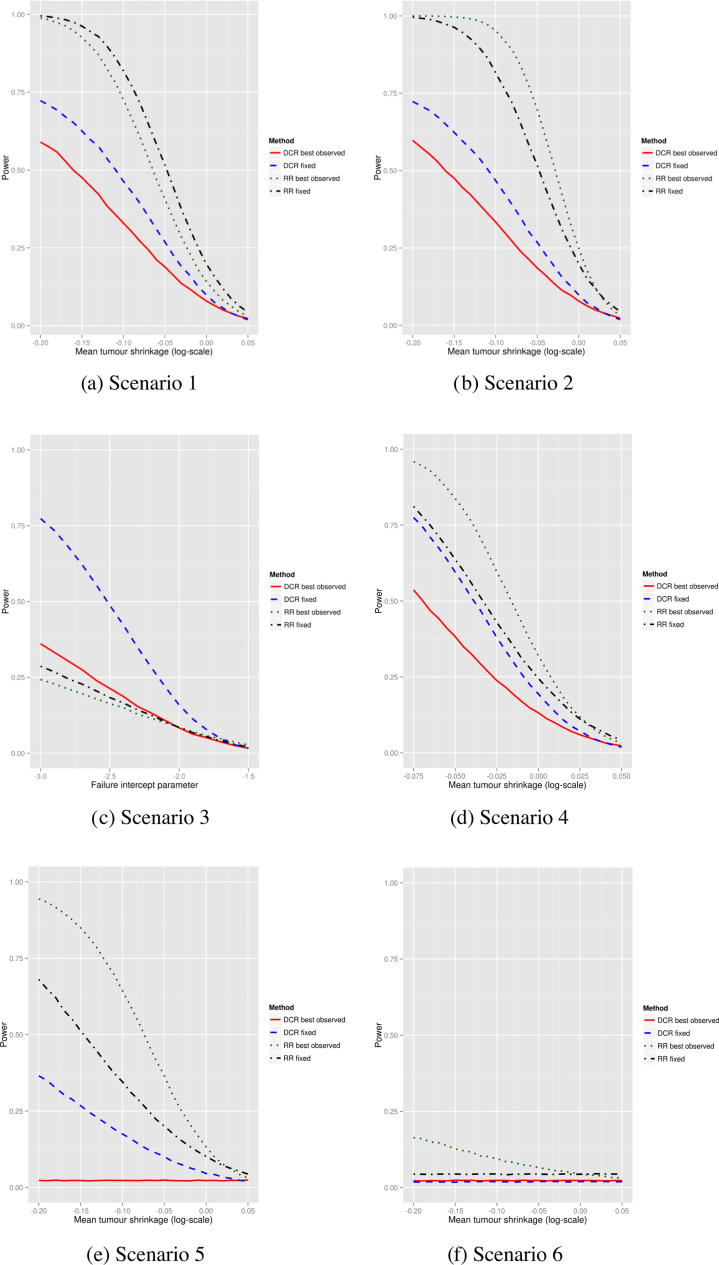
Power of the four response-based end-points for six different scenarios in the one-arm trial setting. Measurement error variance is set to 0. Mean tumour shrinkage is described further in the supplementary material – negative values indicate average shrinkage in the tumour size. The failure intercept parameter in scenario 3 is the parameter that determines the probability of progressing for a non-growth reason. More highly negative values mean lower probabilities of progressing for non-growth reasons.

Fig. 1 shows the power of the four single-arm phase II end-points in the six different scenarios without measurement error. In all cases, 30 patients were simulated, and there was assumed to be no measurement error. In scenario 1 (Fig. 1a), the two RR end-points perform markedly better than the two DCR end-points. Assessing response at a fixed time after treatment was more powerful than assessing the best observed response. Scenario 2 (Fig. 1b) shows that for RR, the best observed response is more powerful. The relative performance of the two DCR end-points remains the same which appears counterintuitive. However both DCR end-points effectively only use the first two non-baseline measurements – in the best observed DCR case, if the one of the first follow-up observations is a progression, then the treatment is a failure, otherwise it is a success. In scenario 3, the two DCR end-points are the most powerful, with fixed considerably more powerful than best observed. In scenario 4 the pattern is the same as in scenario 2 but with less of a gap between the RR and DCR end-points. In scenarios 5 and 6, the power tends to be lower than scenario 2. For scenario 6, the best observed RR only reaches 15% power when the mean tumour shrinkage is high; the other end-points have no power as they are based on the first two follow-up times, in which there is no effect of the experimental treatment.

**Fig. 2:**
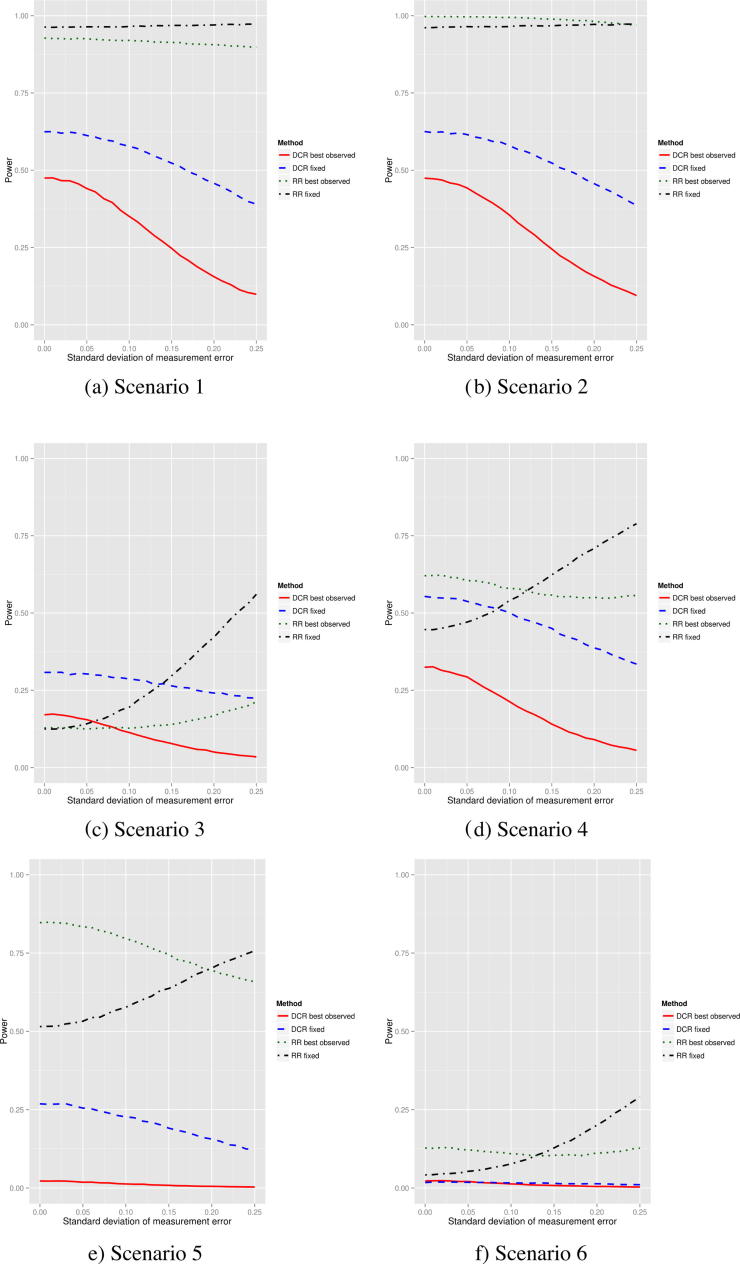
Power of the four response-based end-points for six different scenarios as the measurement error varies in the one-arm trial setting. For scenarios 1, 2, 5 and 6 the mean tumour shrinkage is set to −0.15. For scenario 3, the failure intercept parameter is set to −3. For scenario 4, the failure intercept parameter is set to −2.25 and the mean tumour shrinkage is set to −0.015.

Fig. 2 shows the effect on the power when there is measurement error. The measurement error standard deviation (see supplementary methods for more details) is varied between 0 and 0.25, corresponding to no and extremely high measurement error respectively. Generally the best observed RR end-point is the least affected across the different scenarios. The two DCR end-points generally lose power as the measurement error increases. The power of the fixed RR end-point increases as the measurement error increases. This is partly explained by the type-I error rate. Table 2 shows this (for all scenarios) for three different measurement error levels. When there is no measurement error, all error rates are slightly lower than the 5% level on average. When there is a medium amount of measurement error, the fixed RR end-point has the highest type-I error rate (7.6%). All other end-points appear to control the rate well. When there is an extremely high measurement error, the type-I error rate of the fixed DCR end-point is about right, but the others are inflated, particularly the fixed RR end-point.

### 3.2 Two-arm studies

**Fig. 3:**
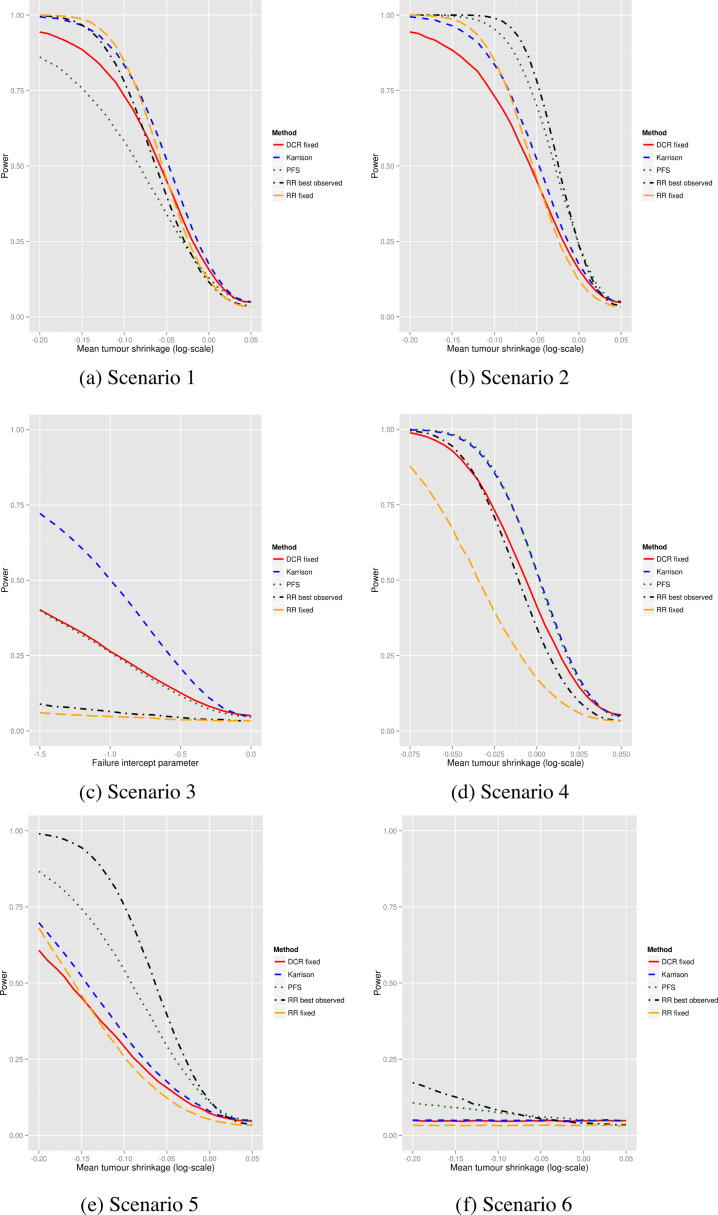
Power of fixed disease control rate (DCR), fixed response rate (RR), best observed RR, progression-free survival (PFS) and Karrison’s method in the two-arm trial setting. Measurement error variance is set to 0. Mean tumour shrinkage is described further in the supplementary material – negative values indicate average shrinkage in the tumour size. The failure intercept parameter in scenario 3 is the parameter that determines the probability of progressing for a non-growth reason. More highly negative values mean lower probabilities of progressing for non-growth reasons.

Fig. 3 shows the comparison of power between RR fixed, RR best observed, DCR fixed, PFS and Karrison’s method for two-arm trials. In all scenarios, 75 patients per arm were simulated. No single end-point performs well in all scenarios. PFS performs well in scenarios 2, 3, 4 and 5 (i.e. when there is a sustained effect of the treatment). Best observed RR performs at least moderately well in all scenarios other than 3, and is best in 2 and 5. Karrison’s method performs best in scenario 3 and well across all scenarios except 6. Fixed RR performs best in 1, but not well in others. Fixed DCR is never best but performs moderately well in 1, 3 and 4. Scenario 6 again has low power for all end-points. Supplementary Fig. 1 shows the power of the four end-points when the measurement error standard deviation is changed. Increased measurement error affects each end-point fairly equally. Table 2 shows the type-I error rate is well controlled in all cases.

## 4 Discussion

We have shown through simulation studies that the power of the different end-points depends on the scenario and no end-point performs well in all cases. End-points based on using all follow-up times (best observed RR and PFS) perform better when the treatment’s effect persists until progression. If the treatment only provides a benefit for a limited amount of time, the fixed-time end-points are generally more powerful. We have also shown that measurement error affects the type-I error rate of single-arm trials, but not two-arm trials. Single-arm trials will also be affected by variability in the estimated historical control response rates [20].

Two scenarios represented a treatment that provides a delayed effect. This is of increasing interest for immunotherapies. In the case of a long delay, none of the phase II end-points we examined had much power. This is because by the time the treatment effect is observed, some or most patients have already progressed. Response criteria for immunotherapies have been proposed [21], and assessment of these would be of interest for future work.

We have only considered RECIST-based end-points as they are the most frequently used in solid tumour phase II trials. However future work could consider novel end-points such as early tumour shrinkage [22], time to tumour growth [23], especially for scenarios, such as a delayed effect, where RECIST-based end-points performed poorly. Novel methods for improving existing end-points could also be compared [24,25].

A relevant question is how well the different end-points do in predicting the treatment’s success at phase III. A clinician who is interested in running a phase II trial should consider how the treatment is likely to bring about improved long-term survival (if that is the phase III end-point to be considered). Pre-clinical information and trials from drugs in the same class could be used to determine if the effect is likely to be throughout treatment, for a limited time or delayed. The results here then can be used to choose a powerful and robust end-point.

## 5 Role of the funding source

The study sponsor had no role in the study design or analysis.

## Conflict of interest statement

None declared.

## Figures and Tables

**Table 1:** Description of the six simulation scenarios.

Scenario	Description
1	For the first two treatment intervals, patients who are given the experimental treatment experience a lower average growth (or higher average shrinkage) than patients given the control treatment. The experimental treatment provides the same average growth as the control treatment for subsequent treatment intervals. There is no difference in the probability of non-growth progressions between the arms. This represents a cytotoxic drug that is given for a fixed period or a drug that is given until progression but is effective only for a limited time
2	Patients given the experimental treatment experience a lower average growth than patients given the control treatment for the entire set of follow-up times. The relative improvement remains constant throughout the trial. There is no difference in the probability of non-growth progressions between arms. This represents a cytotoxic drug that is given until progression of the patient and is effective throughout the time it is given
3	Patients given the experimental treatment have the same average growth in tumour size as those given the control treatment. The experimental treatment results in a lower probability of non-growth progressions at each timepoint. This represents a cytostatic drug that is given until progression of the patient
4	A combination of scenarios 2 and 3 – the experimental treatment both reduces the average growth of target lesions and reduces the probability of non-growth progressions
5	Patients given the experimental treatment have a lower average growth than patients given the control treatment from the second follow-up time onward. There is no difference in the average growth between baseline and the first follow-up time, and there is no difference in the probability of non-growth progressions between arms. This represents a drug that has a delayed effect, such as an immunotherapy
6	As in scenario 5, but the delay is longer so that the treatment only has effect from the fourth follow-up time onward

**Table 2:** Type-I error rate of the end-points as measurement error varies.

End-point	Type-I error rate		
	No measurement error	Medium measurement error (measurement error standard deviation set to 0.1)	High measurement error (measurement error standard deviation set to 0.25)
*Single-arm trials*			
Disease control rate (DCR) fixed	0.039	0.038	0.043
Response rate (RR) fixed	0.043	0.076	0.295
DCR best observed	0.041	0.043	0.092
RR best observed	0.031	0.034	0.091

*Two-arm trials*			
DCR fixed	0.047	0.047	0.050
RR fixed	0.034	0.035	0.042
RR best observed	0.036	0.035	0.038
PFS	0.048	0.048	0.045
Karrison	0.050	0.049	0.051
