# Supplementary material for: The power of phase II end-points for different possible mechanisms of action of an experimental treatment
Source: Eur J Cancer. 2015 May;51(8):984–92. doi: 10.1016/j.ejca.2015.03.002 (PMC4435668; doi:10.1016/j.ejca.2015.03.002)
Supplement: Supplementary data 1 — Supplementary materials. [file mmc1.docx]

**Supplementary methods**

**Technical description of simulated data**

In the main paper, the method of simulating data is described in non-technical language. In this section we provide further technical detail on the simulation process and the simulation scenarios we considered.

For a given treatment arm, we denote by $Y_{ij}$the sum of longest diameter of the target tumour lesions (henceforth referred to as tumour size) of patient i at follow-up time j. The baseline tumour size for patient i is denoted by $Y_{i0}$. A total of N patients are recruited to the treatment arm, so i varies between 1 and N. We assume that patients are followed up for a maximum of J timepoints, so that j varies between 0 and J.

In addition we denote by $F_{ij}$the indicator for whether a non-growth progression occurred for patient i between timepoint j-1 and j. In this case i varies between 1 and N, j varies between 1 and J.

For the simulation studies, the true value (as opposed to the value observed with measurement error) $Y_{ij}$is generated in the following way:

1. $Y_{i0}$, the baseline tumour size for patient i is simulated from a Uniform(1,10) distribution.
2. $\log\left(\frac{Y_{i1}}{Y_{i0}}\right)$, the log tumour-size ratio between the baseline observation and the first follow-up observation is simulated from a $Normal(\mu_{1},\sigma^{2})$ distribution.
3. For the remaining follow-up timepoints, the tumour size is generated by simulating $\log\left(\frac{Y_{ij}}{Y_{i(j-1)}}\right)$ from a $Normal(\mu_{j},\sigma^{2})$ distribution.

Relevant parameters here are the values for $\mu_{1},\mu_{2},\ldots,\mu_{J}$ and $\sigma^{2}$.

Once the true tumour size is simulated, measurement error is added to represent the observed tumour size. For each value of $Y_{ij}$, the observed value is simulated as $Z_{ij}=\exp(\log(Y_{ij})+\varepsilon_{ij})$, where $\varepsilon_{ij}\sim N(0,\sigma_{m}^{2})$. Here, $\varepsilon_{ij}$are independent, identically distributed normal variables with variance $\sigma_{m}^{2}$, which represents the degree of measurement error. The measurement error is added on the log scale as it is likely that the absolute error in measurement would be larger for larger tumour sizes. The values for $Z_{ij}$ are taken as the observed tumour sizes, and are used to classify the patients as successes or failures by the various endpoints.

To simulate the $F_{ij}$indicators, we use logistic regression models:

In this case, the value of $\alpha$is the baseline log-odds of non-growth progression for a patient with a measured tumour size of 0, and $\beta$ is the parameter representing the effect of the tumour size. In all simulation scenarios, we set $\beta$ to 0, so that the probability of non-growth failure is equal for all patients, regardless of their tumour size.

For two-arm studies, patients from the two arms are simulated separately, and parameters are allowed to differ between arms.

**Simulation scenarios**

The simulation scenarios we consider in the main paper are summarised below.

In scenario 1, the parameters for the experimental treatment are set so that $\mu_{3}=\mu_{4}=\mu_{5}=\mu_{6}=0.05$, and $\sigma^{2}=0.25^{2}$, $\alpha=-2,\beta=0$. The values of $\mu_{1}$ and $\mu_{2}$ are set to be equal and are allowed to vary. The variance in the measurement error term, $\sigma_{m}^{2}$also may vary. Subfigures 1a and 3a in the main paper show the power of the endpoints when $\mu_{1}$ and $\mu_{2}$vary from 0.05 to -0.2. In the one-arm case, the null response probabilities are calculated under the case that $\mu_{1}$ and $\mu_{2}$ are equal to 0.05 and there is no measurement error. In the two arm case, the control arm parameters are set to $\mu_{1}=\mu_{2}=\mu_{3}=\mu_{4}=\mu_{5}=\mu_{6}=0.05$, $\sigma^{2}=0.25^{2}$, $\alpha=-2,\beta=0$. The measurement error variance in the control arm is set to be equal to the value in the experimental arm.

In scenario 2, the parameters are set so that $\sigma^{2}=0.25^{2}$, $\alpha=-2,\beta=0$and all $\mu_{j}$are equal and allowed to vary. The variance in the measurement error term, $\sigma_{m}^{2}$also may vary. Subfigures 1b and 3b in the main paper show the power of the endpoints when the $\mu_{j}$ parameters vary from 0.05 to -0.2. The null response probabilities for one-arm trials and the control arm parameters are the same as in scenario 1.

In scenario 3, the parameters are set so that $\mu_{1}=\mu_{2}=\mu_{3}=\mu_{4}=\mu_{5}=\mu_{6}=0.05$, $\sigma^{2}=0.25^{2}$ $\beta=0$. This time the $\alpha$parameter is varied, with subfigures 1c and 3c showing $\alpha$ varying from -1.5 to -3. The null response probabilities for one-arm trials are calculated under the case that $\alpha$=-1.5. For two-arm trials, the control arm parameters are set to $\mu_{1}=\mu_{2}=\mu_{3}=\mu_{4}=\mu_{5}=\mu_{6}=0.05$, $\sigma^{2}=0.25^{2}$, $\alpha=-1.5,\beta=0$.

In scenario 4, the parameters are set so that all$\mu_{j}$are equal and allowed to vary as in scenario 2. The $\alpha$ parameter is also varied as in scenario 3. The values of the$\mu_{j}$vary from 0.05 to -0.75 and the $\alpha$ parameter varies between -1.5 and -2.25. Both parameters are varied at the same time.

In scenario 5, the parameters are set so that $\mu_{1}=0.05,\sigma^{2}=0.25^{2},\alpha=-2,\beta=0.$ The values of $\mu_{2,}\mu_{3},\mu_{4},\mu_{5},\mu_{6}$ are set equal and vary from 0.05 to -0.2. For two-arm trials, the control arm parameters are set as in scenarios 1 and 2.

In scenario 6, the parameters are set so that $\mu_{1}=\mu_{2}=\mu_{3}=0.05,\sigma^{2}=0.25^{2},\alpha=-2,\beta=0.$ The values of $\mu_{4},\mu_{5},\mu_{6}$ are set equal and vary from 0.05 to -0.2. For two-arm trials, the control arm parameters are set as in scenarios 1 and 2.

The standard deviation of the measurement error, $\sigma_{m}$ is varied between 0 and 0.25.A value of 0.05 represents the situation where 95% of observations of tumour size being between 90% and 110% of the actual tumour size. A value of 0.1 gives 95% of observations between 82% and 121% A value of 0.25 gives 95% of observations between 61% and 163% of the actual tumour size.

**Karrison’s method**

We test the difference in $\log\left(\frac{Y_{i2}}{Y_{i0}}\right)$ between the two arms, as described in Karrison et al. (1). For patients who suffered a non-growth progression up to the second time-point, their $\log\left(\frac{Y_{i2}}{Y_{i0}}\right)$ is set to the worst value seen amongst other patients (i.e. the maximum). The differences are tested using a Wilcox rank-sum test.

**Supplementary results**

Supplementary figure 1 – power of fixed DCR, fixed RR, best observed RR, PFS and Karrison’s method as the measurement error varies in the two-arm trial setting. For scenarios 1, 2, 5 and 6 the mean tumour shrinkage is set to -0·15. For scenario 3, the failure intercept parameter is set to -3. For scenario 4, the failure intercept parameter is set to -2.25 and the mean tumour shrinkage is set to -0.015.


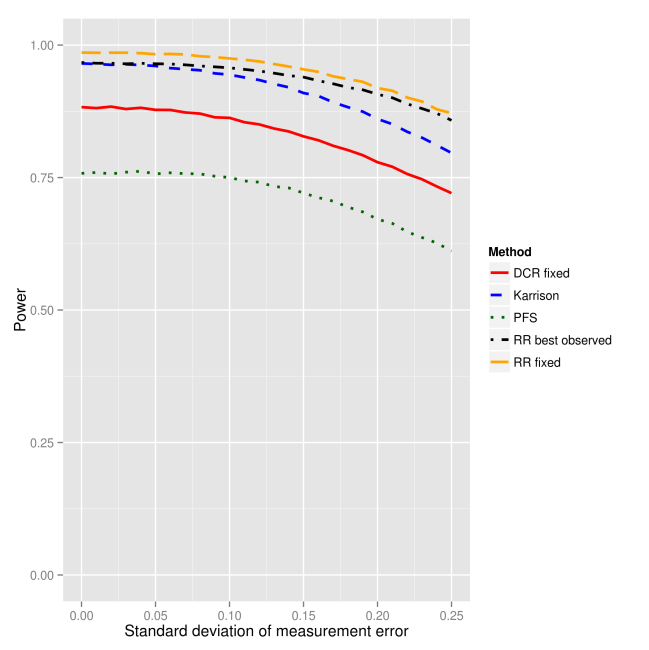

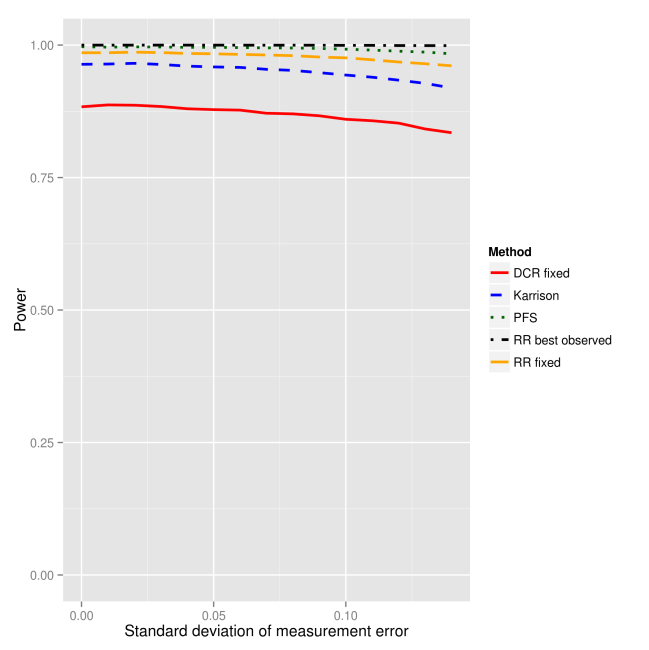


a) Scenario 1 b) Scenario 2


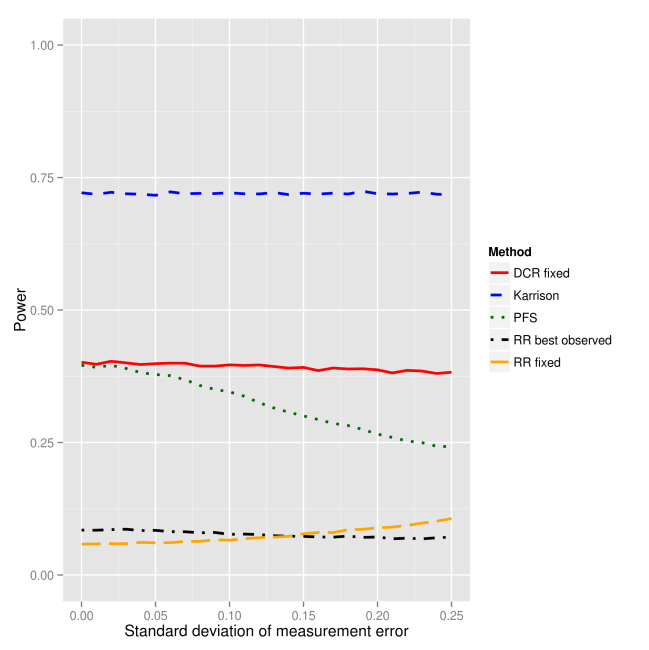

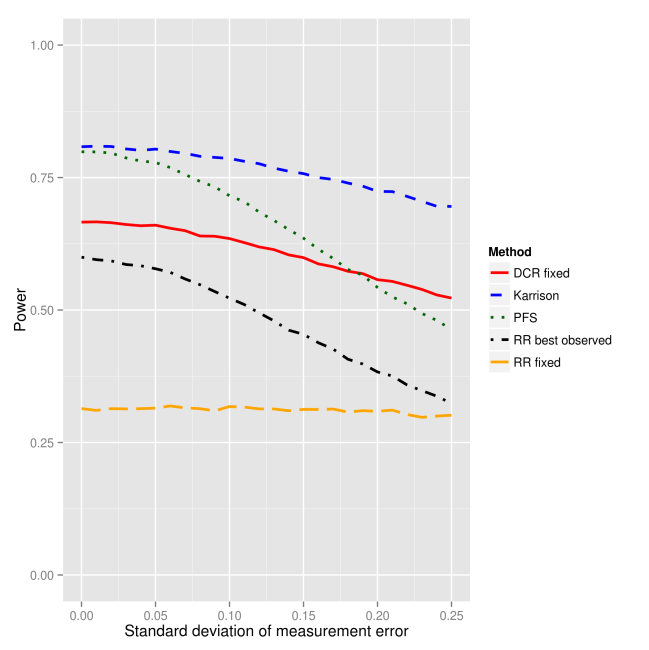


c) Scenario 3 d) Scenario 4


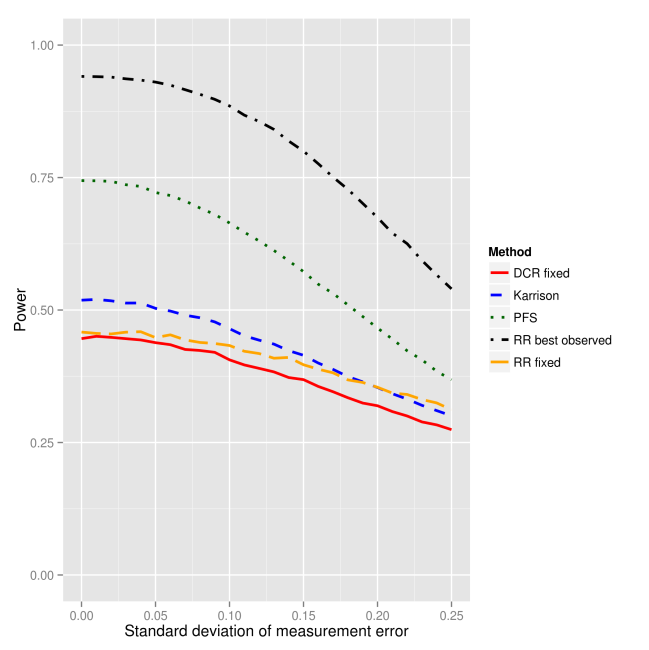

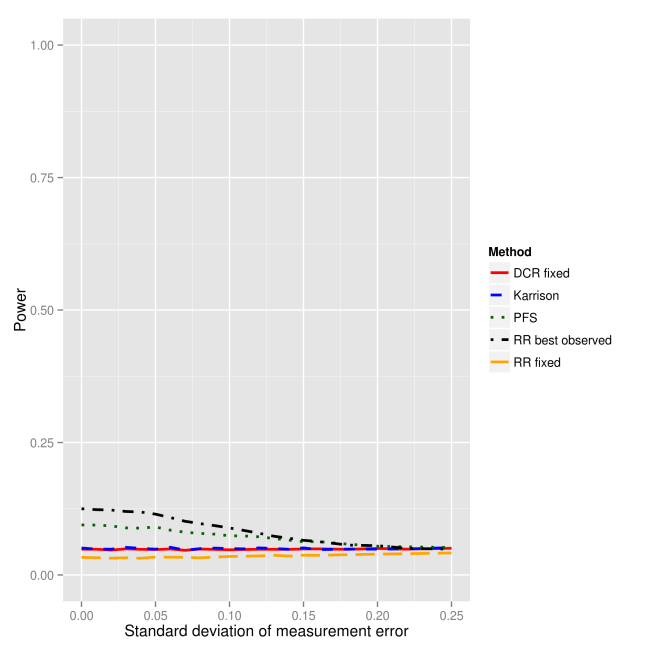


e) Scenario 5 f) Scenario 6

Reference List

(1) Karrison TG, Maitland ML, Stadler WM, Ratain MJ. Design of phase II cancer trials using a continuous endpoint of change in tumor size: application to a study of sorafenib and erlotinib in non-small-cell lung cancer. *Journal of the National Cancer Institute* 2007,**99**(19), 1455-1461.
